# Neoadjuvant chemotherapy impacts axillary lymph node positivity in early breast cancer (cT1-2N0M0) with negative axillary lymph nodes at diagnosis

**DOI:** 10.3389/fmed.2025.1672369

**Published:** 2025-10-10

**Authors:** Jinxiu Ma, Zhimin Fan, Zhifang Jia, Xiaoxiao Dong, Jian Sun, Xiaozhen Wang

**Affiliations:** ^1^Department of Breast Surgery, General Surgery Center, The First Hospital of Jilin University, Changchun, Jilin, China; ^2^Division of Clinical Epidemiology, The First Hospital of Jilin University, Changchun, Jilin, China

**Keywords:** breast cancer, neoadjuvant chemotherapy, sentinel lymph node biopsy, axillary lymph node dissection, invasive disease-free survival

## Abstract

**Purpose:**

This study compared the role of neoadjuvant chemotherapy (NAC) followed by surgery vs. upfront surgery for avoiding axillary lymph node dissection (ALND) in patients with cT1-2N0M0 breast cancer and clinically negative axillary lymph nodes (LNs) at diagnosis.

**Patients and methods:**

Medical records of a sample of 1,695 patients with a primary diagnosis of axillary LN-negative early-stage breast cancer who underwent surgical treatment for breast cancer at the First Bethune Hospital of Jilin University between June 2019 and December 2022 were retrospectively reviewed. The positive rate of sentinel lymph nodes (PR_SLN_) and the positive rate of total axillary lymph nodes (PR_TLN_) were compared between patients who received 4–8 cycles of NAC followed by surgery (*n* = 135) and patients who underwent upfront surgery (*n* = 1,560).

**Results:**

15 patients who received NAC followed by surgery and 79 patients who underwent upfront surgery had positive SLNs. Four patients who received NAC followed by surgery and 1 patient who underwent upfront surgery had other positive LNs. Overall, NAC followed by surgery significantly lowered PR_SLN_ and PR_TLN_ compared to upfront surgery in patients with cT1-2N0M0 breast cancer. In subgroup analyses, PR_SLN_ and PR_TLN_ were significantly lower for NAC followed by surgery compared to upfront surgery in patients aged 40–60 years, with cT2 stage disease, and HER2+ breast cancer. At a median follow-up of 23.15 months, invasive disease-free survival was similar for all patients.

**Conclusion:**

NAC may reduce the rate of axillary LN positivity and the likelihood of ALND in patients aged 40–60 years with cT2N0M0 HER2+ breast cancer and clinically negative axillary LNs at diagnosis.

## Introduction

As of 2020, female breast cancer surpassed lung cancer as the most commonly diagnosed cancer worldwide, with an estimated 2.3 million new cases in 2020, accounting for 11.7% of all cancer cases (1). Breast cancer is a major public health concern. It is a leading cause of morbidity and mortality in most countries and is responsible for one quarter of all cancer cases and one sixth of cancer deaths among women (1).

Diagnostic staging and molecular subtype are critical factors associated with survival in patients with breast cancer. In China, 76.8% of women with breast cancer are diagnosed at Stages I and II (2). The pathological phase of breast cancer is determined by lymph node (LN) involvement and the number and location of positive LNs (3). Patients with localized breast cancer at diagnosis have a 5-year survival rate of 98.8%, whereas patients with a diagnosis of regional breast cancer have a 5-year survival rate of only 85.8% (4).

Currently, breast cancer treatment is focused on de-escalation. Most patients with early-stage breast cancer (cT1 ~ 2N0M0) undergo surgery, with sentinel lymph node biopsy (SLNB) for staging (5). Since 2014, with the publication of results from several clinical trials, including ACOSOG Z0011 (6–8), IBCSG 23-01 (9, 10), AATRM (11), Sinodar One (12), and EORTC 10981-22023 AMAROS (13), SLNB is gradually replacing ALND as the standard surgical procedure for early breast cancer. Neoadjuvant chemotherapy (NAC) and complementary radiotherapy can also minimize the use of ALND; however, only a small proportion of patients with triple-negative (TNBC) or HER2+ breast cancer (tumor >2 cm), or those who have opted for breast-conserving surgery but are contraindicated due to their tumor-to-breast volume ratio, are offered NAC (9). Remarkably, axillary lymph node metastasis remains a possibility in patients with tumor diameters ≤2 cm, with a prevalence ranging from 6 to 31% (14–16); therefore, ALND without radiotherapy is often considered in these cases.

This study investigated the potential role of NAC followed by surgery in patients with cT1-2N0M0 breast cancer and clinically negative axillary LNs at diagnosis. NAC may be beneficial for downstaging disease and improving prognosis, avoiding ALND, and reducing postoperative morbidity, including upper limb edema and long-term shoulder dysfunction, in these patients (17, 18).

## Methods

### Patients

Patients with a primary diagnosis of axillary LN-negative early-stage breast cancer who underwent surgical treatment for breast cancer at the First Bethune Hospital of Jilin University between June 2019 and December 2022 were eligible for this study. Inclusion criteria were: (1) unilateral breast cancer; (2) tumor ≤5 cm in diameter; (3) pre-operative staging with ultrasound and mammography performed at the First Bethune Hospital of Jilin University; (4) pathology revealed invasive breast cancer; and (5) no missing clinical information. Exclusion criteria were: (1) pre-operative staging and/or postoperative pathology performed at a different institution; (2) preoperative diagnosis of axillary LN metastasis; (3) inflammatory breast cancer; (4) previous axillary surgery or radiation therapy to the breast or chest wall; (5) pregnancy or breastfeeding; (6) history of recurrent breast cancer, metastatic breast cancer, or breast cancer accompanied by localized infections; or (7) other malignancies. In this study, a subset of patients was selected for NAC, based on aggressive features (such as HER2+ or TNBC) to reduce recurrence risk and potentially facilitate breast-conserving surgery, as well as strong patient desire for breast conservation despite an initially unfavorable tumor-to-breast size ratio, with the goal of achieving downstaging. These treatment decisions were consistent with guideline. NAC regimens and postoperative therapies were determined according to CSCO guidelines and multidisciplinary team (MDT) discussions (Supplementary Table S5), although a subset of patients did not fully adhere to the recommended treatment. The final analyses included sample of 1,695 patients; of these, 135 patients received 4–8 cycles of NAC followed by surgery and 1,560 patients underwent upfront surgery.

Among all patients, surgical procedures included total mastectomy plus SLNB, total mastectomy plus SLNB and ALND, breast-conserving surgery (BCS) plus SLNB, BCS plus SLNB and ALND, and modified radical mastectomy (MRM). It was determined on clinical and imaging characteristics of the tumor, patient preference, and discussion within MDT. BCS was offered to patients with favorable tumor size and location who provided consent. MRM was performed in cases with multifocal/multicentric disease, tumors not amenable to resection through single incision, inability to achieve negative margins, or upon patient’s preference for mastectomy. For patients initially diagnosed as cN0, axillary management followed SLNB.

Preoperative axillary LN status was evaluated by ultrasonography. Sonographic criteria for determining axillary LN involvement included: cortical thickness (e.g., diffuse cortical thickening >3 mm or eccentric cortical thickening), abnormal morphologic characteristics (e.g., dysmorphic lymph nodes without normal structures, non-gated flow patterns), or lack of fatty hilum (19, 20). Assessment of LNs on ultrasound was performed by two physicians experienced in ultrasonography. If axillary ultrasonography was positive, patients underwent fine needle aspiration (FNA) for cytological examination or hollow needle biopsy followed by pathology to determine whether metastases were present. Patients were identified as cN0 if their axillary LNs showed no abnormalities on ultrasonography and/or if a US-guided biopsy gave negative results (21). To rule out distant metastases, all patients underwent baseline evaluations including chest computed tomography (CT) and abdominal ultrasonography. For those with high-risk factors (cT2 stage, TNBC or HER2+, or suggestive clinical symptoms), further imaging with breast magnetic resonance imaging (MRI), bone scintigraphy, or positron emission tomography-computed tomography (PET-CT) was performed as clinically indicated.

As this was a retrospective study, informed consent to participate from individual patients was waived by the ethics committee of The First Hospital of Jilin University (2023-325), and all methods were performed in accordance with the relevant guidelines and regulations.

### Data collection

Data were collected from the medical record system of our hospital. Information included: patient age at diagnosis, tumor size, histological grade, estrogen receptor (ER), progesterone receptor (PR), human epidermal growth factor receptor-2 (HER2) and Ki-67 status, management plan (treatment modality and cycle), surgical procedure, number of SLNs removed, number of positive SLNs, number of non-SLNs removed, and number of positive non-SLNs. Cancer was staged according to the American Joint Committee on Cancer (AJCC), 7th ed. (22), assuming low Ki67 expression if ≤20% malignant cells exhibited Ki67 staining on immunohistochemistry and high PR expression if >20% malignant cells exhibited PR staining on immunohistochemistry.

### SLNB and ALND procedure

SLNB used a dual-tracer method of 1% methylene blue combined with indocyanine green (ICG). First, 0.5 mL 1% methylene blue (2 mL/branch) was injected subcutaneously at 3, 6, 9, and 12 o’clock at the edge of the areola. Two minutes later, ICG (1 mL/branch) was injected in the same way. After the ICG injection, the mammary gland was massaged for approximately 30 s. Eight minutes later, luminous and blue-stained nodes were removed. For patients who required modified radical surgery or ALND, a skin flap of appropriate thickness was raised, the pectoralis minor and pectoralis major muscles were kept intact, and the skin was cut at the patient’s transverse axillary stripe, allowing removal of level I and II LNs in the ipsilateral axilla (*n* ≥ 10). A small number of patients received technetium 99 sulfur colloid + methylene blue or a combination of nanocarbon + methylene blue as a tracer, with intraoperative rapid frozen pathological examination performed on all blue-stained, fluorescent-appearing, nuclear high signal, and nanocarbon-black-stained LNs that were removed during surgery. Data for these patients were not analyzed separately as the sample size was small and all patients in this study were evaluated with dual tracers, such that type of tracer was not expected to impact outcomes.

### Pathology

Biopsied LNs were embedded in paraffin, serially sectioned at 500 μm intervals, and stained with hematoxylin and eosin (H&E). Patients were considered node-positive based on the presence of isolated tumor cell clusters (ITCs) (≤0.2 mm), micrometastases (>0.2 mm-≤2.0 mm), and macrometastases (>2.0 mm).

### Follow-up

Follow-up ended in May 2023. In accordance with clinical guidelines, patients underwent clinical examination, annual mammography, and additional imaging in cases showing recurrence (21). The clinical endpoint was invasive disease-free survival (iDFS), defined as the time from treatment to first occurrence of one of the following events: ipsilateral invasive breast tumor recurrence, ipsilateral local or regional invasive breast cancer recurrence, distant recurrence, contralateral invasive breast cancer, or death from any cause.

### Statistical analysis

Statistical analysis was performed with SPSS v. 26.0. Normally distributed data were reported as mean ± standard deviation, and compared with the *t*-test. Non-normally distributed data were reported as median (25th–75th percentile) and compared with the Mann–Whitney U test. Qualitative data were reported as frequencies and compared with the *χ*^2^ test or Fisher’s exact test.

Baseline characteristics were compared between patients who received NAC followed by surgery and patients who underwent upfront surgery. Logistic regression-based propensity score matching (matching ratio: 1:3, caliper value 0.04) was used to control for confounding due to patient baseline characteristics that may influence decision-making regarding NAC (patient age at diagnosis, T-staging [T1 vs. T2], molecular subtype).

The positive rate of SLNs (PR_SLN_) and the positive rate of total axillary LNs (PR_TLN_) were calculated using the following formula and were compared between patients who received NAC followed by surgery and patients who underwent upfront surgery.


$$1.\;PR_{\mathrm{SLN}}=\frac{\left(\text{number of}\;\mathrm{SLN}\;\text{positive cases}\right)}{\left(\text{total number of cases with SLNB}\right)}\times 100\%$$

$$2.\;PR_{\mathrm{TLN}}=\frac{\left(\text{number of}\;\mathrm{LN}\;\text{positive cases}\right)}{\left(\text{total number of cases}\right)}\times 100\%$$

(where, number of LN positive cases = number of SLN positive cases + number of cases with ALND).

A two-tailed *p* value <0.05 was considered statistically significant.

## Results

### Patient baseline characteristics

**Figure 1:**
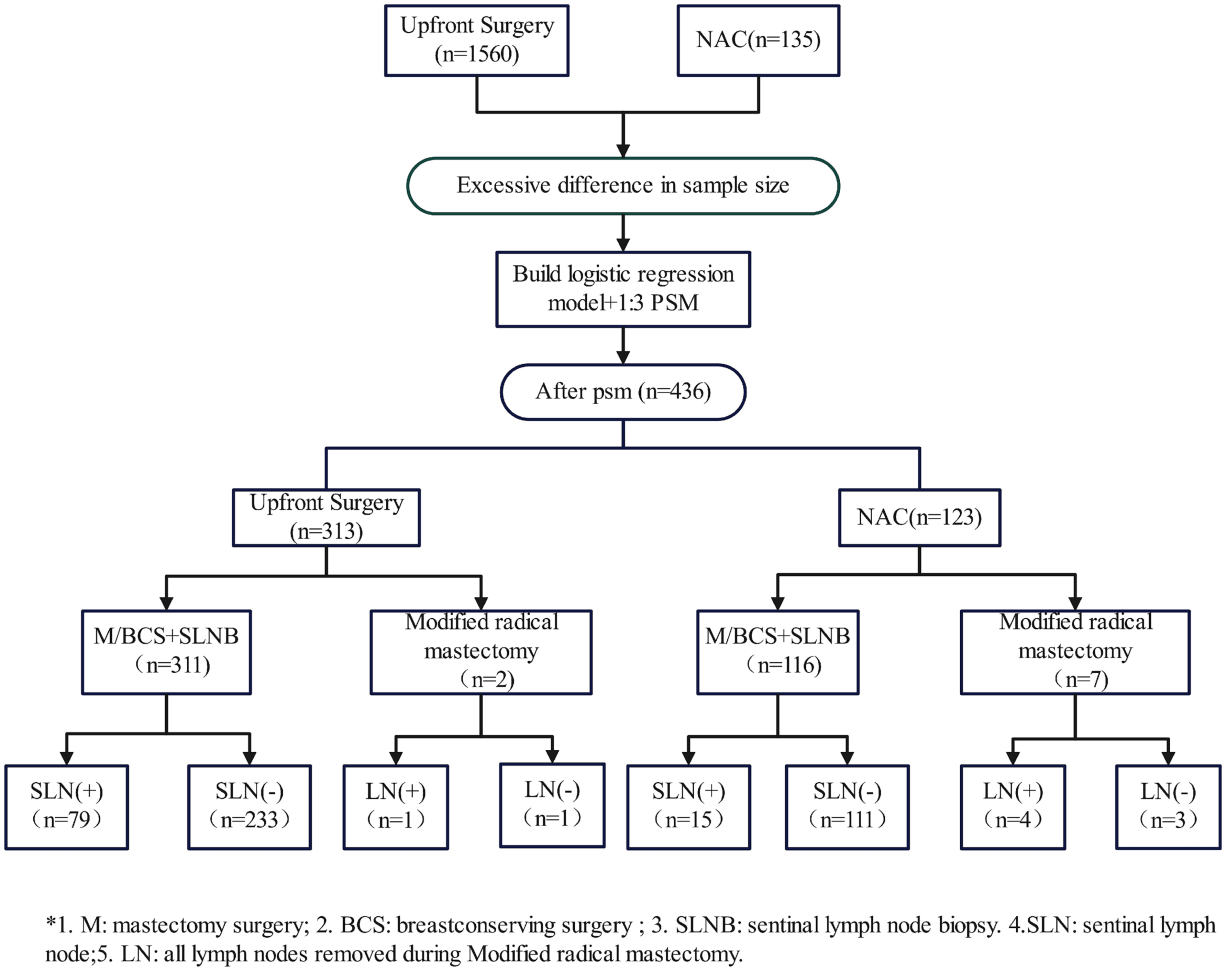
Flow chart of patient selection and propensity score matching.

This study included 1,695 patients; of these, 135 patients received 4–8 cycles of NAC followed by surgery and 1,560 patients underwent upfront surgery. All patients had unilateral breast cancer with no previous history of tumor. Patients who received NAC followed by surgery were significantly younger, had significantly larger tumors and were significantly more likely to have HER2+ or TNBC compared to patients who underwent upfront surgery. As patient age, tumor stage (diameter), and molecular subtype may influence decisions regarding NAC, a logistic regression model was used to balance the baseline characteristics of the two groups of patients (*p* > 0.05). The demographic and clinicopathological characteristics of patients before and after propensity score matching are summarized in Table 1. The number of patients after propensity score matching was 123 who received NAC followed by surgery and 313 who underwent upfront surgery (Figure 1).

**Table 1:** Patient baseline characteristics before and after propensity score matching.

General clinical data		Before			After		
		UpS (*n* = 1,560)	NAC (*n* = 135)	*p*	UpS (*n* = 313)	NAC (*n* = 123)	*p*
Average age (years)		53.24 ± 10.29	51.02 ± 10.09	0.016	51.25 ± 10.94	51.56 ± 9.90	0.786
Tumor size (cm)		21.19 ± 8.34	29.37 ± 9.81	0.000	26.49 ± 9.08	28.29 ± 9.51	0.068
Type	Luminal A	474 (30.38%)	11 (8.15%)	0.000	47 (15.06%)	11 (8.94%)	0.424
	Luminal B	610 (39.10%)	39 (28.89%)		97 (30.99%)	39 (31.71%)	
	HER2+(HR+)	157 (10.06%)	21 (15.56%)		50 (15.97%)	21 (17.07%)	
	HER2+(HR−)	129 (8.27%)	29 (21.48%)		41 (13.10%)	26 (21.14%)	
	TNBC	190 (12.18%)	385 (25.93%)		78 (24.92%)	26 (21.14%)	

## LN positivity

**Figure 2:**
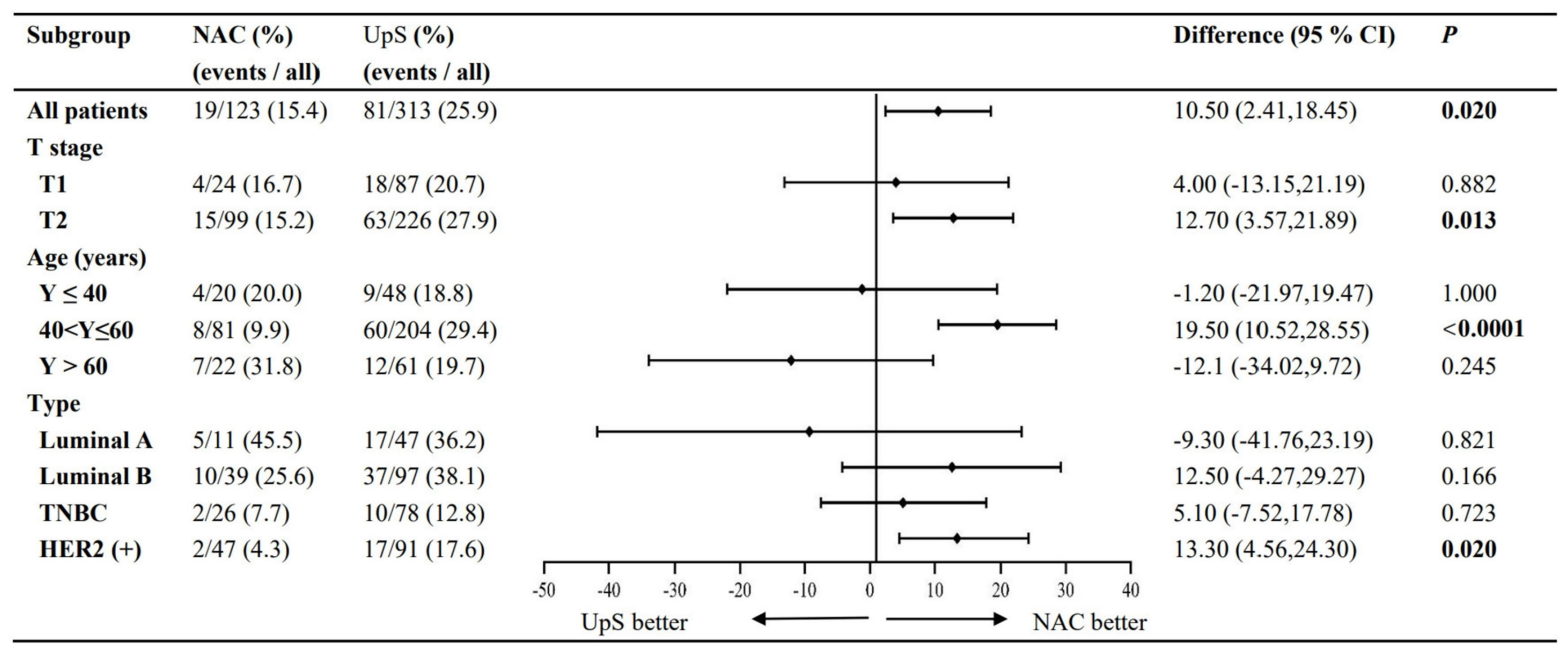
Subgroup analyses: PR_TLN_.

**Figure 3:**
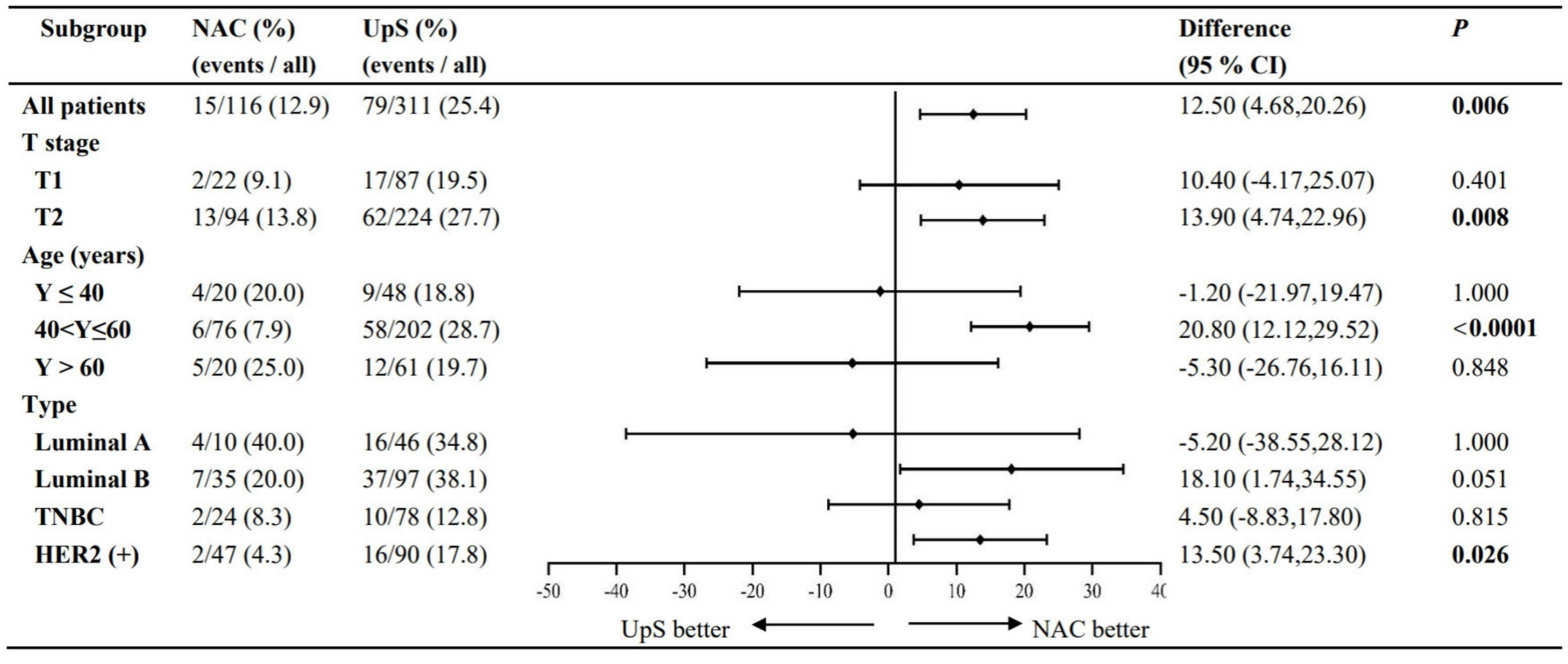
Subgroup analyses: PR_SLN_.

PR_SLN_ and PR_TLN_ are summarized in Table 2. NAC was associated with significantly lower rates of both PR_SLN_ (12.9% vs. 25.4%, RR 0.503, 95% CI 0.302–0.836) and PR_TLN_ (15.4% vs. 25.9%, RR 0.597, 95% CI 0.379–0.940) compared to upfront surgery (Figures 2, 3).

**Table 2:** Subgroup analysis of pCR status and lymph node positivity after NAC.

PR	Group	Total	ypN+ (%)	Difference and 95% CI	*χ* ^2^	*p*
PR_SLN_	B-pCR	57	3 (5.3%)	15.00 [3.28, 26.87]	5.852	0.016
	B-nonpCR	59	12 (20.3%)			
PR_TLN_	B-pCR	58	3 (5.2%)	19.40 [7.52, 31.37]	8.871	0.003
	B-nonpCR	65	16 (24.6%)			

### Subgroup analyses

Subgroup analyses of LN positivity stratified by patient age at diagnosis, cT stage, and molecular subtype were performed for the 436 patients that remained after propensity score matching.

#### Age

Patients were categorized into three groups based on age at diagnosis: ≤40 years (*n* = 48), >40 to ≤ 60 years (*n* = 204), and >60 years (*n* = 61). While NAC significantly reduced both PR_SLN_ and PR_TLN_ in patients aged 40–60 years, the differences were not statistically significant in the ≤40 or >60 age groups, despite a numerical trend toward higher rates with NAC (Figures 2, 3).

#### cT-staging

In accordance with the 7th edition of the AJCC guidelines, tumors were classified as cT1 (≤20 mm; *n* = 111) or cT2 (>20 mm-≤50 mm; *n* = 325) based on maximum tumor diameter before surgery. For cT2 tumors, NAC significantly lowered rates of PR_SLN_ (13.8% vs. 27.7%) and PR_SLN_ (15.2% vs. 27.9%) compared to upfront surgery. A similar, but non-significant, trend was seen in cT1 tumors (Figures 2, 3).

#### Molecular subtype

Tumors were classified as luminal A (*n* = 47), luminal B (*n* = 97), TNBC (*n* = 78), and HER2+ (*n* = 91). A significant reduction in both PR_SLN_ and PR_TLN_ with NAC was observed only in HER2+ breast cancer. For TNBC and Luminal B tumors, NAC showed a numerical reduction in rates, while Luminal A tumors showed a numerical increase; however, these trends were not statistically significant (Figures 2, 3).

#### Pathologic complete response in patients who received NAC

PR_SLN_ and PR_TLN_ in patients who received NAC followed by surgery were investigated further. Patients were stratified based on the presence or absence of breast pathologic complete response (pCR) after NAC, defined as the lack of invasive cancer (ductal carcinoma *in situ* may be present) in the initial breast lesion (Miller-Payne grade 5). PR_SLN_ [5.3% vs. 20.3%, *p* = 0.016, RR = 0.259 (0.077, 0.869)] and PR_TLN_ [5.2% vs. 24.6%, *p* = 0.003, RR = 0.210 (0.064, 0.685)] were statistically significantly lower in patients with breast pCR compared to residual disease after NAC (Table 2). These data imply that the pathological response of a patient’s primary breast lesion to NAC may directly influence axillary LN status.

## Prognosis

### Overall prognosis

**Figure 4:**
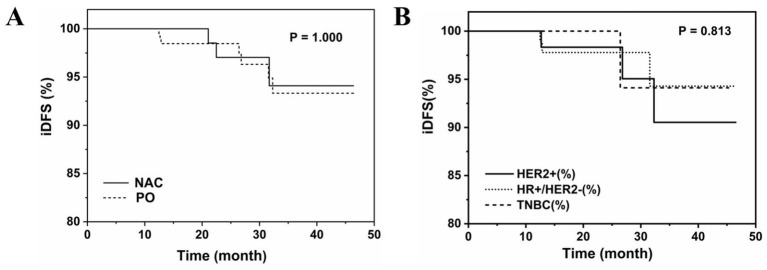
Kaplan–Meier curves **(A)** patients who received NAC followed by surgery or underwent upfront surgery; **(B)** Patients who underwent upfront surgery.

Among all patients, median follow-up was 23.15 months and mean follow-up was 23.85 months. 13% of patients who received NAC followed by surgery and 19.2% of patients who received upfront surgery were lost to follow-up. Three patients who received NAC followed by surgery and seven patients who received upfront surgery developed metastases during follow-up; no other patients experienced recurrence or death (Table 3; Supplementary Table S1). There were no significant differences in the frequency of events determining iDFS for NAC followed by surgery compared to upfront surgery (both 2.8%). Kaplan–Meier estimates of iDFS were 94.09% for NAC followed by surgery and 93.32% for upfront surgery (Figure 4A).

**Table 3:** Clinicopathological characteristics of patients who experienced metastases during follow-up.

Group	Age (year)	Type	Postoperative pathology	Practical treatment	Adverse events
NAC 3 cases	47	TNBC	B: MP2	Pre-o: 8AC-T	Ipsilateral supraclavicular lymph node metastasis at 31.7 months
			L: 0/4	Post-o: none	
	60	Luminal A	B: MP3	Pre-o: 8AC-T	Ipsilateral supraclavicular lymph node metastases at 21.1 months
			L:0/3	Post-o: Letrozole	
	58	TNBC	B: MP2	Pre-o: 3AC+TA+4TP	Brain metastases at 22.7 months
			L:0/5	Post-o: none	
	47	HR-Her-2+	0/2	Failure to comply	Lung metastases, liver metastases, adrenal metastases, pleural metastases at 32.3 months
PO 7 cases	43	Luminal A	1/2 macro ALND 2/15	Failure to comply	Brain metastases at 12.83 months
	72	HR+Her-2+	1/6 macro ALND 0/12	4TCH+Letrozole +17H	Lung metastases at 12.7 months
	35	HR-Her-2+	0/2	8AC-THP+17HP	Breast recurrence at 26.8 months
	48	TNBC	0/5	6TC	Brain metastases at 26.4 months
	74	Luminal B	0/5	Failure to comply	Lung metastases at 12.5 months
	51	Luminal A	2/9	8AC-T	Chest wall recurrence at 31.6 months

B, Breast response; L, Lymph node metastasis; MP, Miller & Payne; A, Anthracyclines; C, Cyclophosphamide; T, Paclitaxel/Paclitaxel-albumin; H, Trastuzumab; P, Perjeta/Platinum; Post-o, post-operative; Pre-o, preoperative.

### Subgroup analyses

#### Pathologic complete response in patients who received NAC

There was no significant difference in the frequency of events determining iDFS between patients with breast pCR (0.0%) compared to residual disease (5.1%) after NAC (Supplementary Table S2).

#### Molecular subtype in patients who received NAC

There were no significant differences in the frequency of events determining iDFS in patients stratified by molecular subtype of breast cancer with breast pCR compared to residual disease after NAC (luminal: 0.0% vs. 3.1%, *p* = 1.000; TNBC: 0.0% vs. 15.4%, *p* = 0.581; HER2+: 0.0% vs. 0.0%) (Supplementary Table S3).

#### Molecular subtype in patients who underwent upfront surgery

There were no significant differences in the frequency of events determining iDFS in patients stratified by molecular subtype of breast cancer who underwent upfront surgery (luminal: 2.54%; TNBC: 1.69%; HER2+: 3.95%) (Figure 4B; Supplementary Table S4).

## Discussion

SLNB, which spares 60–75% of patients from ALND, has become the standard surgical procedure for early breast cancer due to advancements in medical technology and a growing understanding of the disease. Notably, recent large-scale clinical trials have revealed that ALND may not have clinical benefits for patients with early-stage breast cancer (7, 10, 23, 24). However, a considerable portion of patients with axillary LN metastasis still experience a range of post-ALND complications, including sensory abnormalities (25), lymphatic fistulae (26), lymphedema (27), infections (3–15%) (28, 29), hematoma (2–10%) (26) and restricted shoulder joint movement (30).

NAC is widely administered to patients with locally advanced and inoperable breast cancer. Its main purpose is to achieve a pCR (31). The use of NAC in less aggressive breast cancer is more controversial. The present study examined the clinical utility of NAC for patients with early-stage breast cancer (cT1-2N0M0) and clinically negative axillary LNs at diagnosis. Overall, NAC followed by surgery significantly lowered PR_SLN_ and PR_TLN_ compared to upfront surgery in this patient population. In subgroup analyses, PR_SLN_ and PR_TLN_ were significantly lower for NAC followed by surgery compared to upfront surgery in patients aged 40–60 years, with cT2 stage disease, and HER2+ breast cancer. In contrast, patients aged ≤40 years or >60 years or with luminal A cT1-2N0M0 breast cancer were unlikely to achieve axillary downstaging by NAC. Among patients who received NAC followed by surgery, those who achieved breast pCR had significantly lower PR_SLN_ and PR_TLN_ compared to patients with residual disease. Overall, these findings imply that patients with cT2N0M0 HER2+ breast cancer aged 40–60 years may benefit most from NAC followed by surgery through a reduction in the rate of axillary LN positivity and the likelihood of ALND. Our findings align with the current trend in CSCO and NCCN guidelines toward de-escalating axillary surgery in patients who respond favorably to NAC. The significant reduction in nodal positivity rates observed in our study, particularly in HER2+ and cT2 subgroups, contributes to the growing evidence supporting less invasive axillary management. While our data support the avoidance of ALND in initially node-positive patients who convert to ypN0 after NAC, the potential omission of SLNB in those achieving breast pCR remains exploratory. Larger trials (32) are currently investigating this approach, and our results help identify patient subgroups that may be most suitable for such de-escalated strategies in the future.

Previous studies have shown that HER2+ or TNBCs are most likely to be associated with high breast and axillary pCR rates and the possibility of axillary downstaging after NAC. A retrospective study revealed that patients with HR+/HER2+, HR−/HER2+, and TNBC achieved higher breast and axillary pCR rates after NAC than patients with HR+/HER2 breast cancer, and PR_SLN_ was lower (3.6%) in patients with breast pCR (33). Others have reported that patients with cN0 HER2+ or TNBC achieving breast pCR may not need axillary surgery due to an extremely low LN positivity (34, 35).

In the present study, median follow-up was 23.15 months after definitive surgery for primary breast cancer. During follow-up, three patients who received NAC followed by surgery developed supraclavicular lymph node and brain metastases, with a recurrence rate of 2.80% and iDFS of 94.09%. Seven patients who underwent upfront surgery developed recurrent metastases, including bone metastases, lung metastases, *in situ* recurrence of breast cancer, brain metastases, and recurrence in the chest wall, with a recurrence rate of 2.80% and iDFS of 93.32%. Overall, NAC followed by surgery appeared to reduce the risk of tumor recurrence compared to upfront surgery, although the difference was not statistically significant (*p* > 0.05). The principal limitation of this study is its median follow-up of approximately 23.15 months, which is insufficient to draw definitive conclusions regarding long-term survival outcomes. Future studies with extended follow-up are warranted to confirm the oncological safety of this strategy.

In 2023, the Chinese Society of Clinical Oncology (CSCO) updated its guidelines for postoperative intensive treatment in patients with breast cancer who have undergone neoadjuvant therapy (36). In the ExteNET (37) trial, intensive treatment of HER2+ breast cancer with neratinib for 1 year after trastuzumab-based adjuvant therapy led to a 2.5% increase in iDFS. BRCA-related genetic testing is recommended in TNBC with positive LNs and tumors >2 cm. In the absence of mutations, continuous treatment with capecitabine (level of evidence: 2A) for 1 year is recommended, and the five-year iDFS can be improved from 56.1 to 69.8%. If mutations are present, 1 year of continuous olaparib (level of evidence: 1B) can increase the 3-year iDFS from 77.1 to 85.9%. For patients with HR+ breast cancer, the MonarchE (38) study reaffirmed the place of abemaciclib in combination with tamoxifen or an aromatase inhibitor or in the adjuvant treatment of HR+/HER2−breast cancer, especially for adjuvant intensification in high-risk breast cancer (level of evidence: 1A). In the present study, only two patients underwent postoperative treatment intensification with oral capecitabine, one patient underwent postoperative treatment intensification with abemaciclib, and two patients received carilizumab immunotherapy, because of the high cost of CDK4/6 inhibitor or immunosuppressant therapy, among other reasons (Supplementary Table S5). As only a small number of patients underwent postoperative intensive treatment, the impact on iDFS was likely minimal. Beyond clinicopathological factors, genetic profiling—especially BRCA1/2 germline mutation status—plays an increasingly important role in personalized breast cancer treatment. Although genetic testing was not routinely performed for all patients in this study, BRCA status is known to affect surgical choices (risk-reducing mastectomy or oophorectomy) (39), systemic therapy selection (platinum-based chemo or PARP inhibitors) (40), and long-term risk management (41, 42). Future studies incorporating genetic data and NAC response could help optimize axillary management strategies. Larger cohorts and longer follow-up are still needed to better evaluate the effect of treatment intensification on iDFS and OS in early breast cancer.

In conclusion, NAC may reduce the rate of axillary LN positivity and the likelihood of ALND in patients aged 40–60 years with cT2N0M0 HER2+ breast cancer and clinically negative axillary LNs at diagnosis. Improvement in long-term prognosis, upper extremity function, and patient quality of life represent potential benefits of NAC. In the future, clinical trials in NAC should focus on patients with early-stage breast cancer, with findings informing the development of individualized treatment plans that optimize safety and efficacy and prevent overtreatment.

## Data Availability

The datasets presented in this article are not readily available because the datasets generated during and/or analyzed during the current study are not publicly available due to patient’s privacy, but are available from the corresponding author on reasonable request. Requests to access the datasets should be directed to wxzhen@jlu.edu.cn.
