# Supplementary material for: Neoadjuvant chemotherapy impacts axillary lymph node positivity in early breast cancer (cT1-2N0M0) with negative axillary lymph nodes at diagnosis
Source: Front Med (Lausanne). 2025 Oct 10;12:1672369. doi: 10.3389/fmed.2025.1672369 (PMC12549614; doi:10.3389/fmed.2025.1672369)
Supplement: Supplementary file 1 [file Data_Sheet_1.PDF]

Supplementary data

Neoadjuvant chemotherapy impacts axillary lymph node positivity in early breast cancer (cT1-2N0M0) with negative axillary lymph nodes at diagnosis

Group	Validity	Invalidity	Positivity (%)	Difference & 95% CI	χ^2	P
**NAC(n=123)**	107	13.01%	3（2.8%）	0.00	0.000	1.000
**PO (n=313)**	253	19.17%	7（2.8%）	[-0.037, 0.037]		

Table S1. Survival of patients who received NAC followed by surgery or who underwent upfront surgery during the follow-up period

Group	Total（case）	Positivity (%)	Difference & 95% CI	χ^2	P
**B-pCR**	48	0 (0.0%)	5.10	0.992	0.319
**B-nonpCR**	59	3 (5.1%)	[-0.52, 10.69]		

Table S2. Frequency of events determining iDFS in patients with breast pCR or residual disease after NAC

Type	Group	Total	Positivity (%)	Difference & 95% CI	χ^2	P
**Luminal**	B-pCR	10	0 (0.0%)	3.10	0.000	1.000
	B-nonpCR	32	1 (3.1%)	[-2.90，9.15]		
**TNBC**	B-pCR	10	0 (0.0%)	15.40	0.304	0.581
	B-nonpCR	13	2 (15.4%)	[-4.23，34.99]		
**HER2+**	B-pCR	28	0 (0.0%)	0.00	0	/
	B-nonpCR	14	0 (0.0%)	/		

Table S3. Frequency of events determining iDFS in patients stratified by molecular subtype of breast cancer with breast pCR compared to residual disease after NAC

Type	Total	Positivity (%)	χ^2	P
**Luminal**	118	3 (2.54%)		
**TNBC**	59	1 (1.69%)	0.401	0.813
**HER2+**	76	3 (3.95%)		

Table S4. Frequency of events determining iDFS in patients stratified by molecular subtype of breast cancer who underwent upfront surgery

Characteristic	NAC (n = 123)	Prior operation (n = 313)	P
**Age**			0.786
Y<= 40	20	48	
40<Y≤60	81	204	
Y>60	22	61	
**cT**			0.068
T1	24	87	
T2	99	226	
**Biologic subtype**			0.424
Luminal A	11	47	
Luminal B	39	97	
TNBC	26	78	
HER2+	47	91	
**Number of SLNs examined**			
Mean (SD)	6.01	6.26	
Median (range)	4(1-24)	4(1-39)	
**Number of involved SLNs**			
ITC	0	4	
Micrometastasis	2	12	
Macrometastasis	13	63	
**(y) pN**			
N0	105	233	
N+	19	81	
**(Neo)adjuvant Regimen**			
Anthracyclines + taxanes	69	77	
Taxanes	7	44	
Others（including anti-HER2 drugs）	47	50	
**Cycles**			
4	4	28	
6	47	31	
8	69	105	
> 8	2	2	
Others	1	5	
**Intensive treatment after operation**			
AI±OFS	15	95	
CDK4/6i	1	5	
Capecitabine	2	7	
Anti-HER2 drugs	0	0	
Immunotherapy	2	0	

Table S5. Clinicopathological characteristics of patients with cT1~2N0M0 breast cancer who received NAC followed by surgery or who underwent upfront surgery
